# Mass Casualty Decontamination Guidance and Psychosocial Aspects of CBRN Incident Management: A Review and Synthesis

**DOI:** 10.1371/currents.dis.c2d3d652d9d07a2a620ed5429e017ef5

**Published:** 2016-09-27

**Authors:** Holly Carter, Richard Amlôt

**Affiliations:** Emergency Response Department, Public Health England, Salisbury, UK; Emergency Response Department, Public Health England, Salisbury, UK

## Abstract

Introduction: Mass casualty decontamination is an intervention employed by first responders at the scene of an incident involving noxious contaminants.  Many countries have sought to address the challenge of decontaminating large numbers of affected casualties through the provision of rapidly deployable temporary showering structures, with accompanying decontamination protocols.  In this paper we review decontamination guidance for emergency responders and associated research evidence, in order to establish to what extent psychosocial aspects of casualty management have been considered within these documents. The review focuses on five psychosocial aspects of incident management: likely public behaviour; responder management style; communication strategy; privacy/ modesty concerns; and vulnerable groups.

Methods: Two structured literature reviews were carried out; one to identify decontamination guidance documents for first responders, and another to identify evidence which is relevant to the understanding of the psychosocial aspects of mass decontamination.  The guidance documents and relevant research were reviewed to identify whether the guidance documents contain information relating to psychosocial issues and where it exists, that the guidance is consistent with the existing evidence-base.

Results: Psychosocial aspects of incident management receive limited attention in current decontamination guidance.  In addition, our review has identified a number of gaps and inconsistencies between guidance and research evidence.  For each of the five areas we identify: what is currently presented in guidance documents, to what extent this is consistent with the existing research evidence and where it diverges.  We present a series of evidence-based recommendations for updating decontamination guidance to address the psychosocial aspects of mass decontamination.

Conclusions: Effective communication and respect for casualties’ needs are critical in ensuring decontamination is completed quickly and effectively.  We identify a number of areas requiring further research including: identifying effective methods for communicating in an emergency; better understanding of the needs of vulnerable groups during decontamination; effective training for emergency responders on psychosocial issues, and pre-incident public education for incidents involving emergency decontamination.  It is essential that the psychosocial aspects of mass decontamination are not neglected in the pursuit of solely technical solutions.

## Introduction

The likelihood of incidents involving chemical, biological, radiological, and nuclear agents has increased in recent years, due to advances in technology, and the increased willingness of terrorists to use unconventional weapons (1 ^,^ 2 ^,^ 3). While such incidents are still relatively low probability, evidence suggests that if an incident of this type were to occur, it would have a high impact on society. CBRN agents are invisible, consequences of exposure are often unknown, and there is the potential for delayed effects from contamination (4 ^,^ 5 ^,^ 6); they therefore score highly in terms of so-called dread risk (defined by lack of perceived control, catastrophic potential, and fatal consequences) (7), and as such are likely to result in increased public anxiety.

In addition to anxiety caused by the incident itself, it has been suggested that interventions designed to reduce the risk from such agents, such as decontamination, may be more frightening for members of the public than the incident itself, if they are not managed appropriately (8). Decontamination involves those who have potentially been contaminated being asked to remove their clothes (disrobe), and undergo a decontamination process, in order to remove any contaminant from their skin. Traditionally, decontamination has involved showering with soap and water, and in the UK, the New Dimension Mass Decontamination Units were procured for this purpose (9). In recent years, other potential methods of decontamination have been proposed, including dry decontamination with readily available absorbent materials (10), dry decontamination with specially designed absorbent or neutralizing products (11), and decontamination using high air pressure (12). While the number of potential decontamination options has increased in recent years, all of these options will be preceded by some form of disrobe and are likely to be unfamiliar, potentially frightening and embarrassing for members of the public, particularly if they are conducted in a public place. Despite the challenges of managing casualties through decontamination, planning has traditionally focused on technical aspects involved in the decontamination process, with little attempt to understand likely public experiences and behaviour (13). Where public behaviour has been considered there has been a reliance on assumptions about ‘mass panic’, derived from traditional theories of crowd behaviour during emergencies (e.g. 14,15). However, these theories have been discredited by over 50 years of research which shows that crowd members behave normatively and cooperatively in mass emergencies, and that panic is rare (e.g. 16 ^,^ 17 ^,^ 18 ^,^ 19 ^,^ 20 ^,^ 21).

In recent years, research has begun to examine psychosocial[1] aspects of mass decontamination, including public communication needs during mass decontamination (e.g. 22 ^,^ 23 ^,^ 24 ^,^ 25 ^,^ 26), and the impact of mass decontamination on members of vulnerable groups (e.g. 27 ^,^ 28 ^,^ 29 ^,^ 30 ^,^ 31). Such research has provided important insights into how members of the public might experience the decontamination process, and how responder management strategies can impact on public experiences and behaviour during mass decontamination. However, it is not clear whether these research findings have been used to inform guidance documents, training for emergency responders and operational response.

From the research outlined above, it is clear that there are five aspects relating to the psychosocial management of incidents involving mass decontamination which are crucial to the success of the decontamination process. The first is an understanding of likely public behaviour during decontamination. Research suggests that a reliance on common myths about disasters, such as public disorder and mass panic, can impact on the way in which responders manage an incident involving decontamination, and may therefore be crucial to the success of the decontamination process (13). Related to this, the second key psychosocial aspect of incidents involving mass decontamination is responder management style (13 ^,^ 22 ^,^ 23 ^,^ 24). The third is communication strategy, since research has shown that an effective communication strategy can improve both physical and psychological outcomes during mass decontamination (24). The fourth is respect for public concerns about privacy and modesty, which have been shown to increase public compliance, and facilitate the smooth-running of decontamination (25). And the fifth is an understanding of the needs of vulnerable groups, who may require increased assistance in order to undergo decontamination (27 ^,^ 29 ^,^ 30).

The current research sought to review decontamination guidance documents for emergency responders in order to establish to what extent psychosocial aspects have been considered within these documents. The review focuses on the five psychosocial aspects of incident management described above: likely public behaviour; responder management style; communication strategy; privacy/ modesty concerns; and vulnerable groups. Alongside this review of current guidance, we carried out a review of existing literature which is relevant to the understanding of psychosocial aspects of mass decontamination. The guidance documents and relevant research were reviewed to identify: first, whether the guidance documents contain information relating to each of the areas listed above; and second, where information on these issues is included in the documents, whether the guidance is in-line with the existing evidence-base. Following this review, new guidance is suggested for each of the five areas outlined above, based on findings from research. The discussion outlines the main inconsistencies between existing guidance and evidence, and highlights potential areas for further research.

[1] ‘Psychosocial’ includes the psychological and social factors associated with human behaviour, and the interaction between psychological and social factors in any given situation (in this case mass decontamination).

## Method

Two structured literature reviews were carried out, one to identify decontamination guidance documents for first responders, and another to identify evidence which is relevant to the understanding of psychosocial aspects of mass decontamination.

**Literature review of guidance documents**

We used an advanced Google search to identify relevant guidance documents. Several different search terms were generated, including: “mass decontamination” AND “guidance”, “mass decontamination” AND “procedure”, “mass casualty decontamination”, and “decontamination public emergency”. As these search terms often generated over 1,000,000 results, we reviewed the first 200 results from each search. We continued to search for relevant documents until no new documents were identified using our search terms. We reviewed the references of the guidance documents identified from the initial search, which often included references to other relevant guidance documents. We also searched the websites of relevant organisations, including: the UK Fire and Rescue Service, the UK ambulance service, the Federal Emergency Management Agency, the World Health Organisation, and the US Department of Homeland Security (See Appendix 1 for a full list of search terms and results). Inclusion criteria for this search were: documents had to contain guidance on the management of casualties during decontamination; documents had to be designed for use by or to inform the practice of first responders who have some responsibility for managing casualties during mass decontamination (e.g. fire and rescue responders, ambulance responders, hospital staff, the police, the military); and documents had to be national in their area of coverage (we did not include decontamination guidance for individual hospitals, for example), but could be designed to inform the development of local guidance/ protocols. Documents were also limited to those which were publicly available, and which are published in English.

From our literature search, we identified 19 relevant guidance documents. Of these, 9 documents were from the UK (32 ^,^ 33 ^,^ 34 ^,^ 35 ^,^ 36 ^,^ 37 ^,^ 38 ^,^ 39 ^,^ 40). Eight documents were from the US: (41 ^,^ 42 ^,^ 43 ^,^ 44 ^,^ 45 ^,^ 46 ^,^ 47 ^,^ 48). Finally, 1 document was from Australia (49), and 1 was from an international agency (50).

**Literature review of existing research**

We used Google Scholar and PubMed to identify relevant research papers. We generated several search terms, which focused on psychosocial aspects of CBRN incidents generally, and mass decontamination specifically. Search terms included: “mass casualty decontamination”, “CBRN” AND “Psych*”, “CBRN” AND “public communication”, and “public behaviour” AND “decontamination” (see Appendix 1 for a full list of search terms and results). We used the following inclusion criteria: studies had to report original research, including systematic literature reviews; the focus of the research had to be on psychosocial aspects of mass decontamination, or CBRN incidents more generally; studies had to focus on likely public behaviour or responses during an incident; studies had to focus on the psychosocial aspects of the incident(s) for members of the public; studies had to focus on the acute phase of an incident as this is when mass decontamination would take place. We also used the following exclusion criteria: general reviews and opinion articles were excluded; studies relating to psychosocial aspects of non-CBRN emergencies and disasters were excluded; studies examining broader perceptions of terrorism risk were excluded; studies examining the psychosocial aspects of CBRN incidents for emergency responders were excluded; studies which focused on communication strategies or other interventions implemented prior to an incident occurring, or during the recovery phase, were excluded.

Using our search terms, 81 potentially relevant abstracts were identified. After reviewing the abstracts, 32 papers were excluded because they related to procedural aspects of mass decontamination, rather than psychosocial aspects. All remaining 49 papers which were deemed potentially relevant at this stage were reviewed, and the references contained within these papers were also reviewed. Reviewing the references from the initial papers resulted in the identification of a further 34 papers, making 83 papers in total. Following this, 32 papers were excluded because they did not contain empirical research, and a further two papers were excluded because they dealt with individual perceptions of terrorism risk. 49 papers met the inclusion criteria. See Figure 1 for the PRISMA flow diagram for this literature search.

**PRISMA Flow Diagram:**
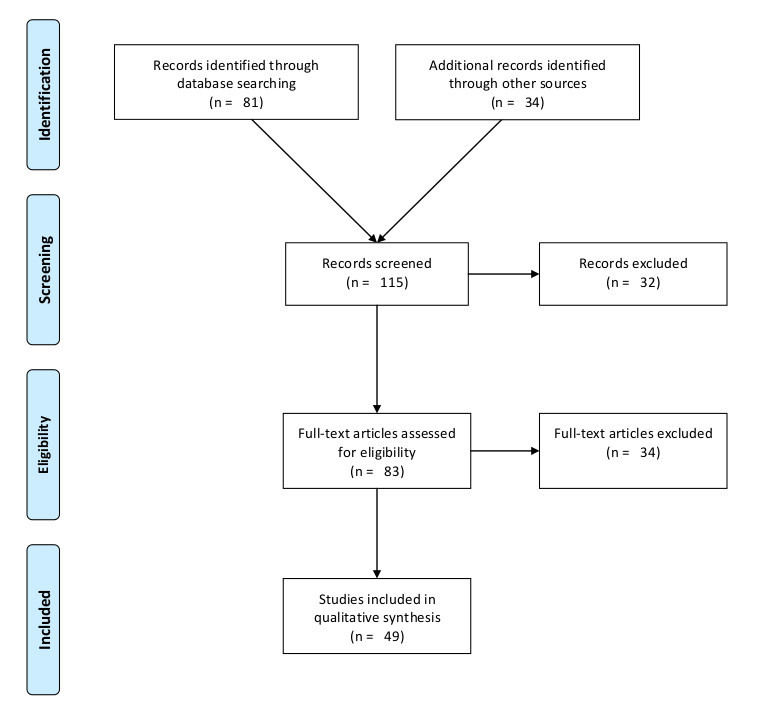

## Results

The results from the review of decontamination guidance documents and available research evidence are presented under each of the five psychosocial aspects of incident management identified in the introduction: likely public behaviour; responder management style; communication strategy; privacy/ modesty concerns; and vulnerable groups. For each of these sections, the results are presented under three headings. First, the information contained within guidance documents is summarised. Second, the available research evidence is summarised. Third, findings from the review of guidance documents and available research are synthesised and used to inform a section on recommendations for updating decontamination guidance documents for emergency responders.

**Likely public behaviour**

*What does the guidance say?*

Ten of the nineteen guidance documents describe how the public are likely to behave during incidents involving mass decontamination. Suggested likely behaviours range from people being confused or frightened and behaving in a disorderly way, to being resilient and behaving in a helpful way.

The most commonly stated likely public behaviour is that a large number of people will be seeking treatment regardless of whether they have been affected by the incident, so-called ‘worried well’. Suggestions include, ‘the number of people seeking treatment will be substantially higher than the numbers exposed or affected (the worried well)’ (36) and ‘expect at least a 5:1 ratio of unaffected to affected casualties’ (47). Linked to this is the suggestion that people who have not actually been exposed to a contaminant may experience psychosomatic symptoms ‘some signs and symptoms may not reflect actual chemical exposure, but manifest as a result of fear, an acute stress reaction, or somatization’ (48).

Other suggested public behaviours can be categorised under themes of orderly behaviour (e.g. people are resilient, do not panic, and will be helpful and cooperative) or disorderly behaviour (e.g. people will be confused and frightened, will behave in a disorderly or aggressive way, and will be non-compliant). Under the theme of orderly behaviour, statements include: ‘expect people not to act quickly and not to panic’ (48) and ‘victims, casualties and bystanders can all provide assistance and wherever possible the emergency services should facilitate self-help at these incidents’ (36). Under the theme of disorderly behaviour, statements in the guidance include: ‘casualties who are on scene may become aggressive in their attempts to be helped’ (46), ‘potential risks to responders include harm from secondary devices, confused, violent, or disruptive casualties’ (37), and ‘the arrival of self-presenting patients may lead to a public order situation which may put staff and/ or patients at risk’ (40). The guidance documents lack clarity on likely public behaviour, and are often inconsistent in their suggestions. Three of the guidance documents which suggest that people are likely to exhibit disorderly behaviours also suggest that people are likely to exhibit orderly behaviours. Three of the guidance documents recognise that the way in which an incident is managed will affect public behaviour. Suggestions about the importance of effective management of members of the public include: ‘clear, credible and timely information during and after the incident will aid order and an efficient response’ (36). However, the lack of evidence to support this position is noted elsewhere: ‘the psychological impact of being exposed to a contaminant is not well studied […] managing crowds, especially crowds who may believe they are in danger is a critical planning factor that needs to be considered and addressed’ (46) (Note: in this paper, [...] is used to represent an abbreviated reference).

Less commonly stated public behaviours include attempts to reunite with family members and loved ones (44), and people leaving the scene but not reporting anywhere for treatment (and thereby potentially causing secondary contamination) (48). Overall, the guidance documents suggest a wide range of different public behaviours, some of which are supported by available evidence, and some of which are not; this is discussed in more detail in the following section.

*What does the evidence suggest?*

While the guidance documents are mixed in their statements as to whether public behaviour will be orderly or disorderly, the available evidence suggests that public behaviour will be orderly. Themes from the review of published evidence include: the public will want and actively seek information (22 ^,^ 23 ^,^ 24 ^,^ 25 ^,^ 26 ^,^ 51 ^,^ 52 ^,^ 53 ^,^ 54 ^,^ 55); people will want to be reunited with family members and loved ones (51 ^,^ 55 ^,^ 56); people will take action to help themselves, their families, and others (22 ^,^ 23 ^,^ 24 ^,^ 25 ^,^ 26 ^,^ 54 ^,^ 55 ^,^ 56 ^,^ 57 ^,^ 58) and people will work together to try to overcome the potential threat (22 ^,^ 23 ^,^ 24 ^,^ 25 ^,^ 26 ^,^ 52 ^,^ 57 ^,^ 59).

Relevant research suggests that incidents involving CBRN agents are likely to score highly in terms of ‘dread risk’, and that this may increase public anxiety (compared to conventional incidents) (54 ^,^ 60). However, while anxiety may be high, it is likely to be proportional to the incident, and people will not panic (59 ^,^ 61 ^,^ 62 ^,^ 63 ^,^ 64). Further, while CBRN incidents may result in increased numbers of ‘worried well’ (although evidence is limited and this term is not considered appropriate: 65), provision of timely and appropriate information should reduce the numbers of people who are ‘worried well’ (65 ^,^ 66 ^,^ 67).

Crucially, a common theme arising from the evidence is that the way in which emergency responders manage the incident will affect the way in which members of the public behave, and this will in turn affect the outcomes of the incident (13 ^,^ 22 ^,^ 23 ^,^ 24 ^,^ 25 ^,^ 26 ^,^ 53 ^,^ 54 ^,^ 68). By contrast, most of the guidance documents do not recognise that public behaviour will vary depending on how the incident is managed, and instead suggest that members of the public are likely to behave in a certain way, and that responder management strategies should be designed to manage this behaviour (i.e. if members of the public are likely to behave in a disorderly or irrational way, they will need to be controlled). One of the documents even lists non-compliant casualties as a vulnerable group, with the clear implication being that if people are behaving in a non-compliant way it is due to individual reasons (e.g. agitation, fear, intoxication), rather than as a result of the way in which the response has been managed (44).

*Updating the guidance*

It is important that guidance documents recognise that potential reasons for non-compliance are many and varied; it is likely that anyone can behave in a non-compliant way under the right circumstances. It is important for guidance to highlight that ineffective management strategies (such as withholding information, or refusing to respect public needs for privacy) may lead to reduced compliance, due to perceptions that responders are behaving in an illegitimate way (13 ^,^ 22 ^,^ 23 ^,^ 24 ^,^ 25).

The lack of consistent understanding about likely public behaviour within the guidance documents is of concern, as research shows that beliefs about public behaviour can impact on responder management strategies during mass decontamination (13 ^,^ 69). Specifically, a belief that the public are likely to panic and behave in a disorderly way during mass decontamination can result in attempts to ‘control’ members of the public, rather than communicate with them. This is as a result of believing that if people are going to behave irrationally, there won’t be any point in trying to communicate with them, and they will need to be ‘controlled’. Ironically, a lack of effective communication could actually create the very disorder which responders are hoping to prevent (24 ^,^ 25 ^,^ 26). As a result of the representation of public behaviour in current guidance documents, and an appraisal of the available evidence, the following recommendations are suggested for new guidance, or revisions to existing guidance:

- Recognise that the majority of members of the public will behave in an orderly way during incident involving decontamination, and that panic will be rare.
- Recognise that likely public behaviours during decontamination include:
- People will want and will actively seek information.
- People will want to be reunited with their families as soon as possible.
- People will take whatever actions they think are appropriate to try to help themselves and others; it is important that people are given sufficient information to enable them to take the most appropriate actions (e.g. moving away from the source of contamination, disrobing, undergoing decontamination etc).

**General management strategies**

*What does the guidance say?*

Guidance documents were reviewed to identify the type of management strategy they advocated. Specifically, we looked for any evidence that guidance documents recommended a ‘control’ management strategy (e.g. statements that members of the public would need to be controlled, stating that information should be withheld) or a ‘respect’ management strategy (e.g. stating that the public should be respected, recommending actions to protect people’s rights). Of the 19 documents identified, only 3 highlight the importance of showing respect for casualties (33 ^,^ 37 ^,^ 38). Of these, NARU (2014) (38) provides the most detailed information, stating that values include: ‘respecting people’s right to privacy, showing people the same respect you would want shown to yourself or a member of your family […] addressing patients in an appropriate manner’.

In contrast, 10 of the guidance documents specifically make reference to the need to ‘control’ casualties during the decontamination process (33 ^,^ 36 ^,^ 37 ^,^ 40 ^,^ 41 ^,^ 42 ^,^ 43 ^,^ 45 ^,^ 46 ^,^ 49). Examples of statements relating to the control of casualties include: ‘Gaining control of victims is a difficult task, but rapidly gaining control is critical to getting victims to quickly perform the critical first step in mass decontamination’ (41), ‘If force is needed to control unruly casualties, use the standing Use of Force guidance established by Incident Command’ (46), and ‘Contain and control casualties to prevent dispersion or re-entry’ (49). Of the 10 documents which suggest that casualties will need to be controlled, 3 state that physical methods can be used to control casualties, including the use of barriers (40 ^,^ 41), and use of water spray to hold people at bay (46).

*What does the evidence suggest?*

The emphasis on control, rather than respect, is unsurprising, given that several of the guidance documents state that members of the public are likely to behave in a disorderly or aggressive way. However, attempts to control members of the public during mass decontamination are likely to be at best ineffective, and at worst, counterproductive.

The review of evidence revealed that control strategies are not recommended for managing CBRN incidents, or decontamination specifically. There are two reasons for this: first, control management strategies are likely to be difficult to implement, due to the small ratio of responders to members of the public, and difficulties in quickly erecting barrier systems (70); second, and perhaps more importantly, control management strategies are likely to be counterproductive, and may actually create the very disorder which they are designed to prevent (24,25,26). Research studies involving mass decontamination exercises and experiments reveal that control management strategies result in casualties not identifying with responders, who are perceived as acting illegitimately. This in turn results in reduced public compliance and cooperation (24 ^,^ 25 ^,^ 26), which may result in delays during the decontamination process, and therefore have a detrimental effect on health outcomes following the incident. In real life incidents, such control management strategies could be even more counterproductive, and could actually lead members of the public to unite to challenge the authority of emergency responders (13).

*Updating the guidance*

It is therefore of concern that the majority of the guidance documents (10 out of 19) recommend a control management strategy. Instead, research suggests that the emphasis should be on showing respect to casualties, through communicating effectively, treating people as individuals, and respecting public needs throughout the process (22 ^,^ 23 ^,^ 24 ^,^ 25 ^,^ 26 ^,^ 57). In particular, effective communication is essential for promoting compliance and cooperation during decontamination, and is an aspect which is often overlooked, particularly if emphasis is placed on controlling casualties (24 ^,^ 25 ^,^ 26). The following recommendations are suggested to revise existing guidance:

- Do not withhold information. Communicate with members of the public through the incident, ensuring that sufficient information is provided to enable members of the public to understand why certain actions are necessary, and to take appropriate actions.
- Communicate updated information as and when it becomes available.
- Demonstrate respect for public needs (e.g. privacy, warmth, shelter) during decontamination, by ensuring that public concerns are listened to, and, where possible, accommodated. Where it is not possible to accommodate public needs, explain why this is the case; never attempt to force someone to undergo decontamination.
- Understand that people have different needs during decontamination, and treat everyone as an individual.

**Communication strategies**

*What does the guidance say?*

Guidance documents were reviewed to establish whether they contained any information about why effective communication with casualties is important. It is important to provide this information within the guidance, because understanding why communication is important is likely to encourage responders to strive to communicate effectively. However, only 7 of the 19 guidance documents explain why effective communication is important (33 ^,^ 36 ^,^ 37 ^,^ 38 ^,^ 43 ^,^ 44 ^,^ 48). Reasons given as to why communication is important include: it will help to reduce public fear and anxiety (36 ^,^ 43 ^,^ 44 ^,^ 48) e.g. ‘to lessen the victims’ fears about the emergency process […] First Responders need to communicate pertinent information that is simple and understandable’ (43); it will promote a more efficient response (36 ^,^ 43 ^,^ 44 ^,^ 48) e.g. ‘clear, credible and timely information during and after the incident will aid order and an efficient response’ (36); it will promote trust and confidence in emergency responders (33 ^,^ 37 ^,^ 38 ^,^ 44) e.g. ‘[Effective communication] will foster public trust and confidence in responding organisations’ (37); and it will reduce non-compliance (37 ^,^ 43 ^,^ 44) e.g. ‘Lack of knowledge among patients may […] potentially increase non-compliance’ (44).

Fifteen of the 19 guidance documents provide some guidance on what should be communicated to members of the public during mass decontamination. The guidance documents suggest that members of the public will need to know: why decontamination is necessary (43 ^,^ 44 ^,^ 45 ^,^ 48 ^,^ 33 ^,^ 36); what to expect during the process (43 ^,^ 44 ^,^ 48); what actions emergency services are taking, and how these will help (33 ^,^ 37 ^,^ 38 ^,^ 43); simple instructions on how to undergo decontamination (33 ^,^ 37 ^,^ 38 ^,^ 41 ^,^ 42 ^,^ 43 ^,^ 44 ^,^ 45 ^,^ 46 ^,^ 48 ^,^ 49); and follow-up information on actions to take and/ or symptoms to look out for (42 ^,^ 43 ^,^ 48 ^,^ 50). Most of the guidance documents (11 out of 19) emphasise the need to provide instructions to members of the public on how to undergo mass decontamination. By contrast, only 6 out of 19 guidance documents state that members of the public should receive an explanation about why decontamination is necessary, and only 4 guidance documents state that members of the public should be informed about the actions emergency responders are taking, and how these will be beneficial. The emphasis within the guidance documents is therefore on providing practical instructions to members of the public, rather than any broader communication about why decontamination is necessary, and what it will involve.

Guidance documents vary widely in the amount of guidance they provide, with amount of guidance on communication ranging from one sentence to several pages of in-depth advice on what to communicate. For example, HPA (2008) (34) simply states: ‘Explain what you are going to do before you start and as you go along’. In contrast, OES (2006) (43) and USDHS (2014) (48) describe in much more detail what should be communicated to members of the public, how it should be communicated, and different methods of communication. OES (2006) (43) also provides some likely questions members of the public may have and possible answers to them. Both of these documents highlight that the way emergency responders communicate (in terms of transparency, consistency, respect, recognition of public concerns, etc.) is as important as what is communicated. Specifically, these two documents recognise that developing an effective communication strategy, including all the factors listed above, will promote increased compliance with the decontamination process and will facilitate more positive outcomes.

Decontamination poses several issues in terms of how information should be communicated to those affected. The main issue relates to the personal protective equipment which emergency responders will be required to wear – this makes it difficult for responders to hear, and be heard by, those affected. Communicating essential information is therefore very difficult, and consideration must be given to how these difficulties can be overcome. This is recognised by 9 of the guidance documents, and potential solutions are suggested: pictograms with step-by-step instructions (33 ^,^ 41 ^,^ 42 ^,^ 43 ^,^ 44 ^,^ 46); pre-recorded audio or video messages (43 ^,^ 44 ^,^ 49); public address system or bullhorn (33 ^,^ 41 ^,^ 46 ^,^ 49); pre-prepared flyers (43 ^,^ 49); instruction cards (33 ^,^ 43); emergency responders acting as ‘guides’ (45). The most common suggestion is to provide brightly coloured pictograms with step-by-step instructions, and place these at the start of the decontamination process, and in the decontamination shower. However, 10 of the guidance documents do not make any suggestions about how to communicate information to members of the public during decontamination.

*What does the evidence suggest?*

Findings from research suggest several reasons why effective communication is important, including: it reduces public confusion and enables appropriate actions to be taken (22 ^,^ 24 ^,^ 57 ^,^ 67 ^,^ 71 ^,^ 72 ^,^ 73 ^,^ 74); it promotes trust in and perceived legitimacy of responders and officials (24 ^,^ 25 ^,^ 60 ^,^ 64 ^,^ 68 ^,^ 69 ^,^ 74 ^,^ 75 ^,^ 76); it promotes a sense of control and reduces public anxiety (23 ^,^ 26 ^,^ 55 ^,^ 58 ^,^ 60 ^,^ 64 ^,^ 72 ^,^ 77 ^,^ 78); and it promotes increased public compliance (22 ^,^ 23 ^,^ 24 ^,^ 25 ^,^ 26 ^,^ 52 ^,^ 60 ^,^ 68 ^,^ 74 ^,^ 75).

For example, Carter et al. (22 ^,^ 23 ^,^ 24 ^,^ 25) analysed the findings from a series of emergency preparedness field exercises and experiments involving mass decontamination, and found that effective communication from emergency responders was crucial for improving both physical outcomes (speed and efficiency of decontamination) and psychological outcomes (increased public compliance, reduced public anxiety). Importantly, Carter et al. suggest that for communication to be effective, it must be initiated as soon as possible; emergency responders should not wait until all information is known before initiating communication with members of the public. The reason for this is that delayed communication can result in reduced perceptions of the legitimacy of responders’ actions, which can in turn result in reduced identification with emergency responders. For communication and information to be beneficial, it is important that those receiving the information (members of the public) identify with those providing the information (emergency responders). If identity is not shared between information providers and information receivers, the information provided will likely be meaningless and will not promote a positive public response. It is therefore essential that emergency responders initiate communication with members of the public immediately (in order to promote perceptions of responder legitimacy and foster shared identification between themselves and members of the public), and continue to communicate consistently throughout the incident.

There is a wealth of evidence which can be used to understand what information should be communicated to members of the public before, during, and after mass decontamination. Information needs in the initial phase of a CBRN incident (prior to undergoing mass decontamination) include: the likelihood of exposure (51 ^,^ 56 ^,^ 65 ^,^ 76 ^,^ 79); what actions can be taken to prevent or minimise exposure (e.g. decontamination) (22 ^,^ 23 ^,^ 24 ^,^ 25 ^,^ 26 ^,^ 51 ^,^ 54 ^,^ 55 ^,^ 56 ^,^ 59 ^,^ 64 ^,^ 67 ^,^ 68 ^,^ 73 ^,^ 74 ^,^ 76 ^,^ 77 ^,^ 80); why certain actions (e.g. decontamination) are necessary, in terms of the health benefits (24 ^,^ 26 ^,^ 29 ^,^ 53 ^,^ 60 ^,^ 63 ^,^ 73 ^,^ 76 ^,^ 80); and how health interventions, such as decontamination, can protect someone and their loved ones (24 ^,^ 26 ^,^ 76). Information needs during mass decontamination include: practical information about what to do during the decontamination process (e.g. where to go, when to enter the decontamination shower, when to begin rerobe etc.) (22 ^,^ 23 ^,^ 24 ^,^ 25 ^,^ 26 ^,^ 30 ^,^ 76); and specific instructions about actions to take (e.g. instructions about how to wash in the decontamination shower, instructions on how to disrobe) (22 ^,^ 24 ^,^ 25 ^,^ 30 ^,^ 57). Information needs after mass decontamination include: whether interventions or treatments have been effective (29 ^,^ 75 ^,^ 76 ^,^ 79); and information about possible psychological reactions to look out for (58).

As well as highlighting specific information which should be communicated, there are general factors relating to effective communication, which are applicable at all stages of an incident involving mass decontamination: information must be consistent (53 ^,^ 59 ^,^ 60 ^,^ 68 ^,^ 75 ^,^ 81); information should be delivered in a timely way – do not withhold information until all the facts are known (24 ^,^ 26 ^,^ 64 ^,^ 67 ^,^ 76); honesty is key – if information is not known, this should be explained, and updates given when further information becomes available (24 ^,^ 26 ^,^ 53 ^,^ 55 ^,^ 63 ^,^ 65 ^,^ 68 ^,^ 74 ^,^ 75 ^,^ 80 ^,^ 82); demonstrate respect and empathy for those affected (22 ^,^ 23 ^,^ 24 ^,^ 25 ^,^ 26 ^,^ 63 ^,^ 68 ^,^ 74); and avoid unsubstantiated reassurance (53 ^,^ 76 ^,^ 79).

Several studies have specifically considered different ways to communicate with members of the public during mass decontamination, with efficacy being measured in terms of increased removal of contaminant (83), increased speed and efficiency of decontamination (69 ^,^ 80), and improved psychological outcomes (22 ^,^ 29 ^,^ 30 ^,^ 69). Various methods of communication have been suggested, including provision of pictorial instructions (22,29,30,57,83), demonstration of the decontamination process by first responders (57 ^,^ 69), and use of pre-recorded messages delivered over loudspeaker (69). Findings from these studies suggest that both pictorial instructions and demonstration of the process by first responders will result in improved speed and efficiency of decontamination, and improved psychological outcomes (22 ^,^ 29 ^,^ 30 ^,^ 57 ^,^ 69). However, findings relating to the effectiveness of pictorial instructions for improving physical efficacy of decontamination were less conclusive. Amlôt et al., (2010) (83) found that the provision of pictorial instructions did not appear to increase people’s ability to wash effectively during the decontamination process. A possible reason for this was that pictorial instructions were provided prior to, rather than during, decontamination, and were therefore not visible to people as they went through the decontamination shower (30 ^,^ 83). As this is the only study which has examined the impact of different methods of communication on physical efficacy of decontamination, this requires further research.

As well as the studies examining communication methods during mass decontamination specifically, several studies have also examined communication methods during CBRN incidents generally. A key finding arising from these studies is that the person or organisation communicating with members of the public is as important as the message(s) communicated (68 ^,^ 75 ^,^ 76 ^,^ 82). In a series of focus groups with postal workers and Senate employees following the anthrax attacks in 2001, Blanchard et al., (2005) (75) found that information was perceived as being more trustworthy (and people were more likely to act on information given), if it came from a trusted spokesperson. This trusted spokesperson should have some level of authority which enables them to provide trustworthy health information; this can either be because they are a health expert (56 ^,^ 74 ^,^ 76 ^,^ 77), or because they are seen as being representative of the group of people affected (e.g. local officials), or are familiar to them in some way (e.g. family member or friend) (56 ^,^ 68 ^,^ 74 ^,^ 75 ^,^ 76 ^,^ 77). Findings from a mass decontamination field trial reveal that this may be because information is more trusted to the extent that those receiving the information (members of the public) identify with those giving the information (emergency responders) (24).

During an incident involving decontamination, constraints around staff availability (and the need for staff to wear PPE), will limit the choice of who can provide information to members of the public. One possible option is for emergency responders to engage an ambulant casualty to communicate information to other casualties. Research has shown that casualties are willing to help each other during decontamination (22 ^,^ 24 ^,^ 25 ^,^ 26 ^,^ 57), and there is no reason to think that this help would not extend to providing information and guidance to others. Evidence for the effectiveness of using casualties to demonstrate procedures is limited, and available evidence suggests that having a casualty demonstrate necessary actions to others may slow the process down (57). However, this finding was based on two self-reported observations and so may not be reliable. Further, a potential reduction in the speed of demonstration (it may be more time-consuming to explain to a casualty what to demonstrate than for emergency responders to demonstrate actions themselves), should be weighed up against the potential for improved adherence to procedures for safe disrobe of contaminated clothing. As reported above, it is widely recognised that information provided by someone who is representative of the group affected (e.g. another casualty) is more likely to be trusted (and therefore promote compliance) than information provided by those in authority. Given the limited availability of people who could potentially provide information during incidents involving mass decontamination, the idea of asking a casualty to provide information and guidance to others is one that is worthy of further research.

*Updating the guidance*

Seven of the 19 guidance documents provide information about the importance of effective responder communication, and the information provided within these 7 guidance documents is broadly in-line with the research findings described above. However, 12 of the 19 guidance documents do not explain why communication is important. Further, while evidence suggests that one of the most important functions of effective communication is to increase trust in and perceived legitimacy of responders (because this will increase public compliance), this is only recognised by four of the 19 guidance documents (33 ^,^ 37 ^,^ 38 ^,^ 44). Failure to explain why effective communication is important may lead to a belief among emergency responders that effective communication is not essential, or even that effective communication with crowds during mass decontamination is not possible (84).

An effective communication strategy will therefore be essential during incidents involving CBRN agents, and during mass decontamination specifically. For a communication strategy to be effective, it must include information on why communication is important, what should be communicated, appropriate communication style, and methods for communicating information. It is essential that the importance of an effective communication strategy is recognised, and this is seen as a key aspect of successful management of an incident involving decontamination, and that an effective communication strategy is initiated rapidly and continues consistently throughout the incident.

However, only two of the guidance documents contain information on all these factors, and therefore only these two documents can be said to contain a communication strategy (43 ^,^ 48). While 9 other documents do contain some information on communicating with members of the public during mass decontamination, it is limited, and often amounts to only a couple of sentences or a paragraph. Further, these guidance documents tend to focus on practical instructions which should be provided to members of the public, rather than on broader explanations such as why decontamination is necessary, how it will be beneficial, and what actions responders are taking and why; however, evidence suggests that these broader explanations are likely to be as, if not more, important.

While practical information will be necessary during decontamination, broader explanation and rationale are likely to be needed before decontamination – if this is not provided, people may not comply with the need for decontamination in the first place. It is important to recognise a distinction between willingness to comply (promoted by health-focused explanations about the need for decontamination, and explanations about actions responders are taking) and ability to comply (promoted by practical instructions); promoting both willingness and ability to comply will be essential for the smooth-running of the decontamination process.

Based on our review of available evidence, several recommendations can be made for effective responder management strategies for mass decontamination. These recommendations can be divided into three sub-headings: why communication is important; what responders should communicate to members of the public; and how responders should communicate with members of the public. The following recommendations are suggested to revise existing guidance:

Why is communication important?

Communicating effectively is vital in order to facilitate the smooth-running of an incident involving mass decontamination. There are several reasons for this, including:

- Effective communication increases perceptions of legitimacy of the response, and enhances trust in emergency responders.
- Effective communication reduces public confusion and enables members of the public to take appropriate actions during decontamination.
- Effective communication promotes a sense of control, and therefore reduces public anxiety about decontamination.
- Effective communication results in increased public compliance.
- It is important that effective communication is initiated immediately; do not wait until all the facts are known before initiating communication with members of the public. This is important, because communicating effectively from the start of an incident will help to foster a sense of shared identity between responders and members of the public; if this shared identity is not present from the outset, any later attempts at communication may be ineffectual and/ or not trusted.

What to communicate?

- In the initial phase of an incident (prior to mass decontamination):
- Communication with members of the public prior to them undergoing decontamination should focus on providing a rationale for decontamination, to enable people to understand why decontamination is necessary. Specific information which members of the public will want to know prior to undergoing decontamination includes:
- The likelihood that they have been exposed to a contaminant.
- What actions they can take to prevent or minimise their exposure to the contaminant; decontamination is one such action.
- Why decontamination is necessary, in terms of the health benefits of undergoing decontamination.

- During mass decontamination:
- Provide sufficient practical information to allow members of the public to successfully undergo mass decontamination. Such information includes:
- Practical information about what to do during the decontamination process. Such information includes, but is not limited to:
- When members of the public should begin disrobe prior to undergoing decontamination.
- Where members of the public should go during decontamination.
- When members of the public should enter and exit the decontamination shower.
- When members of the public should rerobe.

- After mass decontamination
- The main piece of information which members of the public will want following decontamination is information about whether decontamination has been effective.
- It will also be beneficial to provide members of the public with possible psychological reactions of which they should be aware.

- General communication throughout an incident involving decontamination
- Throughout an incident involving decontamination, some general points for ensuring effective communication include:
- Communicate consistent information - make sure that information is consistent across sources, to avoid confusion, and maintain public trust.
- Deliver information in a timely way - do not withhold information until all the facts are known.
- Be honest when providing information - if certain information is not known, this should be explained, and updates should be given when further information becomes available.

How to communicate

- Communicating with members of the public will be physically challenging, because it may be difficult to hear, and be heard by, members of the public. There are several ways in which physical difficulties with communication can be overcome, including:
- Provide pictorial instructions both prior to and during the decontamination process.
- Provide pre-recorded information messages over loudspeaker.
- Provide a physical demonstration of the decontamination process.
- Communicating with members of the public will also be challenging in terms of ensuring that members of the public trust, and are therefore prepared to act on, any information provided. The extent to which members of the public trust the information provided to them is likely to depend on the extent to which they identify with those providing the information.
- As noted under 'Why is communication important?' above, emergency responders can promote shared identification between themselves and members of the public by communication consistently throughout an incident. If shared identity develops, information provided by emergency responders is likely to be trusted by members of the public, who are then more likely to take appropriate actions based on this information.
- It may also be possible to ask members of the public to provide information to others who have been affected. For example, by explaining to one casualty why decontamination is important, and how to undergo decontamination, and then asking them to explain it to others. Potential advantages include:
- The member of the public will not be wearing PPE, and will therefore be heard more easily by other members of the public.
- Members of the public are likely to identify with the person providing the information to them, because they will be in the same position. Information which comes from another member of the public may therefore be trusted more readily.

**Privacy/ modesty concerns**

*What does the guidance say?*

Almost all of the guidance documents (16 out of 19) mention the need to protect public privacy and dignity. The amount of detail provided ranges from one or two sentences e.g. ‘Ensure that […] patients (and staff) have privacy’ (34) to several paragraphs explaining why protection of privacy is important and how this can be achieved (37 ^,^ 43 ^,^ 48). Seven of the guidance documents specify why protecting privacy is important, with reasons including: failure to protect privacy may result in reduced trust in responders (44); failure to protect privacy may result in reduced public compliance (40 ^,^ 41 ^,^ 44 ^,^ 48 ^,^ 49); protecting privacy will improve comfort of the casualty and their family members during decontamination (43 ^,^ 48); and failure to protect privacy may result in embarrassment (46). Twelve of the guidance documents specify potential methods for protecting public privacy and modesty, including: provision of materials/ clothing to provide cover during disrobe and/ or rerobe (33 ^,^ 37 ^,^ 38 ^,^ 41 ^,^ 42 ^,^ 43 ^,^ 44 ^,^ 46 ^,^ 48); curtains to separate lanes within the decontamination shower (44); separate facilities/ areas for males and females (43 ^,^ 44 ^,^ 46 ^,^ 47 ^,^ 48 ^,^ 49 ^,^ 50); provision of cover from bystanders (38 ^,^ 43 ^,^ 48 ^,^ 49); and allowing casualties to keep their underwear on during decontamination (41 ^,^ 48 ^,^ 49).

The guidance documents therefore recognise various methods of protecting public privacy during decontamination. However, protection of public privacy is secondary to ensuring that decontamination takes place as quickly as possible e.g. ‘responding and receiving organisations must balance concerns surrounding patient privacy and the urgency of decontamination needs’ (48); ‘where practical, ensure that patients are treated with dignity and respect’ (40); and ‘[ensure] the welfare and dignity of casualties as far as possible’ (37); these documents fail to realise that if privacy is not protected, people may refuse to comply with decontamination. This is discussed in more detail below.

*What does the evidence suggest?*

Evidence from real incidents involving decontamination, and from decontamination research trials and field exercises, shows that failure to respect public needs for privacy can result in reduced trust in responders, and consequently in reduced public compliance with decontamination (22 24 ^,^ 25 ^,^ 57 ^,^ 58 ^,^ 80 ^,^ 85). Protecting public privacy has been shown to be a key step in promoting perceptions of responder legitimacy (24 ^,^ 25), a factor which promotes increased public compliance and cooperation (24 ^,^ 25 ^,^ 26), and reduced public anxiety (26). Suggested ways of maintaining public privacy include: providing an area where members of the public can disrobe, out of view of onlookers (58); providing modesty facilities in the disrobe area (80); and provide modesty coverings as soon as possible following decontamination (57). It is essential that members of the public are not forced to disrobe (58). This will be humiliating and degrading for those involved, and could result in perceptions of responder illegitimacy, and attempts to challenge the authority of emergency responders (13 ^,^ 24).

*Updating the guidance*

Despite the increasing evidence that protection of public privacy is vital to increase public compliance, only 5 of the 19 guidance documents recognise that failure to respect public privacy may result in refusal to comply with decontamination. Failure to recognise this may result in a perception that protecting public privacy is an optional extra, which can be considered if time and resources allow. Instead, provision for protecting public privacy should be considered as a vital element of the decontamination process; far from creating delays in the decontamination process, protecting public privacy should reduce delays associated with public non-compliance, and should therefore improve the speed and efficiency of the decontamination process (24). The following recommendations are suggested to revise existing guidance:

- It is essential to ensure that members of the public have sufficient privacy during decontamination; failure to do so may result in members of the public refusing to comply with disrobe and decontamination.
- Under no circumstances should members of the public be forced to disrobe. At best, this will be embarrassing or humiliating for those involved. At worst, this could lead to perceptions of illegitimacy, and attempts to challenge the authority of emergency responders.
- There are several ways in which privacy and dignity can be maintained, including:
- Provision of a private area where people can disrobe, out of sight of onlookers.
- Provision of modesty facilities in the disrobe area.
- Provision of modesty coverings as soon as possible following decontamination.

**Vulnerable groups**

In terms of vulnerability during decontamination, it is useful to distinguish between three different types of casualties: ambulant casualties – these are casualties who are not seriously injured as a result of the incident, and are able to successfully undergo mass decontamination with no assistance from other casualties or emergency responders; non-ambulant casualties – these are casualties who are unable to walk, either as a result of injuries incurred during the incident, or because of an existing impairment, and who will need to go through the non-ambulant decontamination process, and be decontaminated by emergency responders; and potentially non-ambulant casualties – these are casualties who have a vulnerability which may make it difficult for them to successfully undergo mass decontamination; these casualties may be able to successfully undergo mass decontamination, provided appropriate support is in place. Decontamination guidance documents typically focus on planning for the management of ambulant and non-ambulant casualties, with only limited attention being given to potentially non-ambulant casualties. This guidance will therefore focus on planning for the management of vulnerable groups, who fall into the potentially non-ambulant category.

There are a large number of different factors which may make someone vulnerable during decontamination, and there is often considerable overlap between the needs of different vulnerable groups (e.g. difficulty in communicating may be experienced by foreign language speakers, children, older adults, those who have impaired hearing, and indeed just about anyone). To provide focused guidance on actions which can be taken to meet the functional needs of different vulnerable groups, the information and recommendations presented here will be based on a functional needs approach. Four main areas of increased functional need may prevent casualties from successfully undergoing self-decontamination: impaired ability to physically undergo decontamination (e.g. any factors which make it physically challenging for individuals to undergo decontamination), impaired ability to communicate during the decontamination process (e.g. any factors which make it difficult for individuals to hear, see, or understand instructions provided), different social or cultural needs (e.g. cultural norms or religious norms), and pre-existing health factors or medical conditions (e.g. any factors which may make people more susceptible to the effects of contamination, or pre-existing conditions for which people need medication). Recommendations will therefore focus on ways to improve the decontamination process for those with different functional needs.

*What does the guidance say?*

The majority of the guidance documents (15 of 19) provide some guidance on managing vulnerable groups during the decontamination process; only four documents do not provide guidance on the management of vulnerable groups (32 ^,^ 35 ^,^ 39 ^,^ 42). There are a wide range of vulnerable groups identified in the guidance documents, including (but not limited to): children, the elderly, pregnant women, non-English speakers, those with visual, hearing, or physical impairment, those with cognitive impairment, those with mental illnesses, those from different social or cultural backgrounds, and those with pre-existing medical conditions . The amount of guidance provided ranges from a brief mention that the needs of vulnerable groups should be considered e.g. ‘Vulnerable individuals will be cared for according to LA Major Incident plans’ (38), to several pages of in-depth discussion about the needs of vulnerable groups (e.g. 48). While it is important for emergency responders to understand the needs of different vulnerable groups, there is considerable overlap between the needs of many of these groups (as noted above), and the information provided within the guidance documents does not always focus specifically on how the functional needs of different groups can be met. The amount of information provided within the guidance documents, and the wide range in vulnerable groups identified, may be overwhelming for emergency responders. It may therefore be more beneficial to provide information related to specific functional needs which people may have during decontamination, and to tailor guidance and recommendations around these.

Various recommendations are made in the guidance documents for assisting members of vulnerable groups. Although not presented in this way within the guidance, it is possible to separate these recommendations into the four specific functional needs highlighted above. Recommendations to assist those with difficulty in physically undergoing the decontamination process include: implementation of a buddy system (33 ^,^ 36 ^,^ 46 ^,^ 48 ^,^ 50); getting parents or guardians to assist children (48 ^,^ 50); provision of additional personnel to assist people in going through decontamination (46 ^,^ 48); process those with physical impairment through non-ambulatory decontamination if necessary (46); allow people to retain functional aids (including service animals) during decontamination (34 ^,^ 44 ^,^ 45 ^,^ 48); reassure those who have functional aids removed from them (33 ^,^ 36); and implement additional training for assisting members of at-risk groups - include such groups in training and exercising (48).

Many of the recommendations which are designed to assist those with physical impairments may also be used to assist those with difficulties in communicating during decontamination. Examples include: implementation of a buddy system; allowing people to retain functional aids, and implementing additional training for members of at-risk groups. Further recommendations which are designed specifically to assist those with difficulty in communicating during decontamination include: when communicating with children, make eye contact and explain clearly what is going to happen (48); consider using cartoon posters/ videos when communicating with children (44); have an English speaker translate instructions for non-English speakers (48); provide simple instructions in multiple languages (44 ^,^ 46 ^,^ 48); provide instructions in pictogram format (36 ^,^ 37 ^,^ 46 ^,^ 48); consider integrating an interpreter into response plans (36 ^,^ 48).

Recommendations to assist those with pre-existing health or medical conditions include: observe carefully those who may be more susceptible to heat or cold (e.g. children, the elderly) (48); use foil blankets to prevent hypothermia (44); prioritise decontamination of infants and children (44 ^,^ 48 ^,^ 49); pay special attention to cardiac patients and the elderly if outdoor wet decontamination is necessary (45); use tepid water to decontaminate infants (49); and give particular consideration to minimising exposure of pregnant women if a radiological release is suspected (36)

Recommendations to assist those with different social or cultural needs include: keep family groups together (36 ^,^ 37 ^,^ 40 ^,^ 44 ^,^ 47 ^,^ 48 ^,^ 50); remain sensitive to different religious and cultural needs (33 ^,^ 36 ^,^ 37 ^,^ 40 ^,^ 43 ^,^ 44 ^,^ 48); ensure proper levels of modesty through separate lines for males and females, and cover from onlookers (34 ^,^ 36 ^,^ 44 ^,^ 48); and provide clothing to protect modesty (42 ^,^ 44)

There are also some general recommendations for assisting those with different functional needs through the decontamination process, including: treat each person as an expert in his or her specific needs (44); ask people about impairments for which they may need further assistance (44); identify at-risk groups within the community during the planning process (48); implement planning, training, and communication with members of at-risk groups prior to an incident (48); and develop response plans which include specific protocols for at-risk patients (48).

The guidance documents therefore make many recommendations on how to improve the decontamination process for those with different functional needs. It should be noted that while the suggestions described above are likely to be particularly beneficial for members of at-risk groups, implementation of these recommendations is also likely to assist all members of the public in undergoing the decontamination process.

*What does the evidence suggest?*

Evidence suggests that reduced mobility makes people feel more vulnerable (55), and it is important to make provision for decontaminating those who may struggle to physically undergo the decontamination process (80). Evidence supports some of the recommendations described in the guidance documents, particularly in terms of the benefit of implementing a buddy system (57 ^,^ 83 ^,^ 86). Monteith (2013) (57) found that a buddy system is particularly beneficial for members of at-risk groups, but is also likely to improve the decontamination process for everyone. It is therefore a key recommendation which can be made to improve the speed and efficiency of the decontamination process. Another key suggestion in the literature is that those who struggle to physically undergo decontamination should be treated with respect, and that a physical disability does not imply a cognitive disability (30). Another potential issue which has been identified in the literature (but which is not included in the guidance documents) is that people may struggle to rerobe following decontamination. Egan and Amlôt (2012) (71) found that the rerobe section of a decontamination unit may create a bottleneck, whereby people have to wait to be decontaminated because people are still rerobing. They suggest that one way to overcome this would be to increase rerobe capacity, such as including an additional rerobe section, so that those who have been decontaminated can use the first rerobe section to dry themselves, and the second rerobe section to get dressed. This was found to improve the speed and efficiency of decontamination. The literature is also clear that responders should ask each person about their needs and the best way these can be met (29 ^,^ 30). This is not only important for ensuring that each person’s needs are met, but is also crucial for ensuring that responders are perceived to be treating people with respect; it is therefore a key recommendation which should be implemented when managing at-risk groups during decontamination. Several guidance documents recommend that those with physical impairment keep their functional aids when going through decontamination, and there is little evidence to suggest whether this would be beneficial. Taylor et al., (2008) (29) found that those with functional aids would be willing to give these up in a life or death situation, but it may actually be beneficial (in terms of improving speed and efficiency of decontamination) to allow people to retain their functional aids.

There are several recommendations in the literature for managing those with difficulties in communicating during the decontamination process. Recommendations include: provision of simple pictorial instructions showing people what to do during the decontamination process (28 ^,^ 30 ^,^ 51 ^,^ 80 ^,^ 86); provision of instructions in multiple languages (30 ^,^ 76 ^,^ 80); use of body/ sign language to communicate with non-English speakers and those who are hard of hearing (30); keep groups of same language speakers together (30); and consider use of interpreters (if they are readily available) (30 ^,^ 75 ^,^ 76). As above, a buddy system has also been shown to be beneficial for enabling those with difficulties in communicating to successfully undergo decontamination (57 ^,^ 83 ^,^ 86). The guidance documents which contain information on managing those with difficulties communicating during decontamination are broadly in-line with the recommendations in the literature, with the most commonly stated recommendations being to initiate a buddy system and to provide pictorial instructions, with both recommended by 4 guidance documents.

Most of the evidence relating to managing those with pre-existing health or medical conditions during decontamination relates to the increased vulnerability of children during such incidents. Evidence suggests that children are especially susceptible to the effects of contamination (58 ^,^ 66), and are also at increased risk of hypothermia (27 ^,^ 31 ^,^ 58 ^,^ 87). Recommendations from the literature therefore include use of heating lamps (58), use of blankets (31) and use of warm water to prevent hypothermia (28 ^,^ 31).

It can be argued that each individual has their own social and cultural norms, and therefore needs, during decontamination. Several of the aspects associated with decontamination (e.g. disrobing and showering in front of others) go against general societal norms, and are therefore likely to result in increased stress for most people. However, for some groups, this may be particularly distressing, and this should be recognised. Recommendations in the literature which deal with understanding and respecting different social and cultural needs are likely to be universally beneficial. Recommendations include: keep family groups together during decontamination (27 ^,^ 28 ^,^ 87); and consider ethical, religious and cultural issues when asking people to disrobe during decontamination – ensure that sufficient privacy is provided (27 ^,^ 28 ^,^ 31 ^,^ 75 ^,^ 80 ^,^ 87). The recommendations in the literature for managing those with different social and cultural needs therefore broadly support the recommendations made in the guidance documents.

Overall recommendations from the literature on the management of vulnerable groups during decontamination relate to treating people with respect, and treating people as experts on their own condition(s) (29 ^,^ 30). This includes asking each person whether they have a disability (some disabilities are not immediately obvious), and including members of vulnerable groups in planning and training for incidents involving decontamination. Many of the recommendations made in the literature for improving the decontamination process for members of vulnerable groups will also improve the process for others. For example, treating people with respect, treating everyone as an expert on his or her own needs, the provision of large pictorial instructions, and implementation of a buddy system should help to improve the decontamination process for everyone. These recommendations should therefore be considered fundamental to any comprehensive guidance document.

*Updating the guidance*

Where information is presented in the guidance for managing vulnerable groups, this is mainly in line with available evidence. However, there are two notable discrepancies. First, some of the guidance documents suggest that parents should assist their children in undergoing decontamination (48 ^,^ 50). However, evidence suggests that parents may struggle to decontaminate themselves and their children, and that hot zone staff should be ready to assist in decontaminating children (27 ^,^ 28). This supports the recommendation in the guidance documents that additional personnel should be available to assist those with difficulty in physically undergoing decontamination (e.g. 46 ^,^ 48). Second, a key recommendation in the evidence is that responders should ask each person about their needs and how these can be met, but this is only noted as a recommendation in two of the guidance documents (44 ^,^ 48).

Of all vulnerable groups, both the guidance documents and literature contain most information on managing children through decontamination. Guidance is broadly in-line with the recommendations in the literature, and tends to focus on the prevention of hypothermia (44 ^,^ 45 ^,^ 48 ^,^ 49). In both the literature and the guidance documents, relatively little information is provided about managing other pre-existing health factors.

Guidance for assisting those with difficulty in physically undergoing self-decontamination

- Those with difficulty in physically undergoing self-decontamination should be assessed, to determine whether they are capable of undergoing self-decontamination (with additional assistance), or whether they need to undergo non-ambulatory decontamination.
- If members of the public are assessed as being capable of undergoing self-decontamination with additional assistance, there are several recommendations which may help them. These include:
- Implement a buddy system, whereby a member of an at-risk group is paired with another member of the public who can help them to physically undergo decontamination.
- Treat those with a physical disability with respect, and ask them about their needs; a physical disability does not imply a cognitive disability.
- Provide additional personnel who are available to assist those with difficulty in physically undergoing decontamination.
- Be aware that those with difficulty in physically undergoing decontamination may be slower to rerobe. Consider the provision of an additional rerobe section to provide more time for people to rerobe.
- Consider whether it may be possible to allow those with difficulty in physically undergoing decontamination to retain any functional aids they may have.

Guidance for assisting those with difficulty in communicating during self-decontamination

- Several recommendations can be made for managing those with difficulties in communicating during the decontamination process. Recommendations include:
- Provide simple pictorial instructions showing people what to do during the decontamination process.
- Provide instruction in multiple languages.
- Use body/ sign language to communicate with non-English speakers and those who are hard of hearing.
- Keep groups of same language speakers together.
- Find out whether there are any bilingual people in the crowd - they may be able to interpret instructions for others.
- Consider use of professional interpreters (if they are readily available).
- Implement a buddy system, whereby a member of an at-risk group is paired with another member of the public who can help them to understand what they need to do during the decontamination process.

Guidance for assisting those with pre-existing medical conditions

- Recognise that there are a variety of health and medical factors which may make some people more vulnerable than others during incidents involving decontamination, either because of increased susceptibility to the contaminant, or because of factors associate with undergoing decontamination (e.g. risk of hypothermia)
- Ask people about any pre-existing medical conditions they may have.
- Supervise closely anyone who may be at increased risk of hypothermia during decontamination (e.g. children, the elderly, cardiac patients). Some recommendations for preventing hypothermia include:
- Use heating lamps to keep people warm during and after decontamination.
- Use warm water to decontaminate people, especially those at increased risk of hypothermia.
- Provide blankets to keep people warm after decontamination.

Guidance for assisting those with different social or cultural needs

- Several of the aspects associated with decontamination (e.g. disrobing and showering in front of others) go against general norms of society, and are therefore likely to result in increased stress for most people. However, for some groups, this may be particularly distressing, and this should be recognised. Recommendations for understanding and respecting those with different social and cultural needs include:
- Keep family groups together during decontamination.
- Consider ethical, religious and cultural issues when asking people to disrobe during decontamination.
- A key issue which may go against religious and cultural values is undressing in front of others. It is therefore crucial that every effort is made to maintain the privacy and dignity of those affected; this is true for everyone, not just those with different religious and cultural values (see section on privacy, above). However, some specific recommendations which may be useful for managing those with different religious and cultural needs include:
- Ensure that members of the public are decontaminated by personnel of the same gender (where possible).
- Give people the option to go through the decontamination process individually, after others have gone through decontamination.

Cross-cutting recommendations which may assist more than one at-risk group

- As well as the specific recommendations for those with different functional needs, there are also some cross-cutting recommendations which can be made which may assist everyone during decontamination:
- Ask people whether they have any special needs during decontamination; don't assume that members of at-risk groups will be immediately obvious.
- Treat everyone as an expert in his or her own needs: ask the person how best you can assist them.
- Implement training and exercising with members of at-risk groups, to ensure that responders have experience of managing those with different functional needs during the decontamination process.

## Discussion

The review of guidance documents reveals that the documents contain some information relating to psychosocial aspects of mass decontamination, such as likely public behaviour, effective communication with members of the public, and respect for public needs for privacy. However, the information within these documents is often limited to one or two sentences, with little recognition that understanding psychosocial aspects of decontamination will be crucial to the success of the decontamination process. The review of evidence reveals that various studies have been carried out into the psychosocial aspects of mass decontamination, and the findings could be used to inform guidance and training for emergency responders. There are several inconsistencies between the guidance documents and the available evidence, and there is therefore a need to update the guidance documents for emergency responders, to ensure that these are based on relevant research.

**Inconsistencies between guidance and evidence**

There are several inconsistencies between the guidance documents and the available evidence. The main areas of inconsistency are likely public behaviour, overall management strategy, and the amount of importance placed on communication; available research evidence shows these factors are essential to successful incident management, but this is not consistently reflected in the guidance reviewed. A key difference between guidance documents and available evidence is the description of likely public behaviour. While evidence overwhelmingly suggests that members of the public will behave in an orderly and cooperative way, this is not reflected in the guidance documents. As noted above, this belief about disorderly public behaviour may well be the reason that the majority of the guidance documents (10 out of 19) highlight the need to control, rather than communicate with, members of the public. Further, regardless of what type of public behaviour they describe, the documents are universal in their suggestion that the public will behave in a certain way, and responder management strategies should be designed to manage this behaviour. In contrast, evidence suggests that the way the public behave will be influenced by the way the incident is managed; thus responder management strategies should be designed to facilitate positive public behaviour (e.g. compliance, cooperation etc), rather than to reduce negative public behaviour (e.g. non-compliance, disorderly behaviour). It is therefore essential that guidance documents are updated to reflect the available evidence, as this will form a basis for moving away from a focus on control of members of the public towards a focus on communicating with members of the public.

**Improving psychosocial guidance and training for mass decontamination**

This review represents a first attempt to synthesise evidence from mass decontamination guidance documents and relevant literature on psychosocial aspects of CBRN incidents, and use this to create improved guidance for emergency responders. There are a large number of decontamination guidance documents for emergency responders, and there is a need to improve consistency, and ensure that all documents contain relevant information on preparing for psychosocial aspects of such incidents. It is hoped that the evidence provided here can be a starting point for informing such guidance and ensuring that psychosocial aspects are included in future guidance documents.

As noted in the review above, improving guidance documents for emergency responders is not enough; provision of training for emergency responders on psychosocial aspects is also essential. Such training should run alongside technical training, and ensure that psychosocial aspects are seen as fundamental to preparing for and managing incidents involving mass decontamination, and are not an after-thought. This will help to ensure that all emergency responders have a good understanding of the importance of considering psychosocial aspects during mass decontamination, and that they are aware of how their own actions, and the ways in which they manage the incident, can impact on public experiences and behaviour.

**Areas requiring further research**

How to communicate information

There is now a wealth of evidence relating to the importance of effective responder communication during mass decontamination, and the type of information which members of the public will need. However, the research into how best to provide this information is limited. This is likely to be a particular problem during incidents involving mass decontamination, because the PPE which responders are required to wear will render verbal communication difficult. Possible suggestions for communicating information to members of the public during decontamination include provision of pictorial instructions, use of casualty volunteers to demonstrate actions, and provision of pre-recorded messages over loudspeaker. However, further research is required to examine which method (or combination of methods) is the most effective for communicating information to members of the public during decontamination.

Understanding the needs of vulnerable groups during decontamination

Research into understanding the needs of vulnerable groups during decontamination is limited, and the research which has been conducted has tended to focus on the needs of children during decontamination (e.g. 27 ^,^ 28 ^,^ 31). Future research is therefore needed in order to understand more about the needs of other vulnerable groups during decontamination. Research is particularly limited in relation to the needs of those with pre-existing health conditions during decontamination. Future research could therefore explore this, in particular any issues around prioritisation of those with different health needs, and identification of those at increased risk from any contaminant. Evidence is also mixed as to whether those with physical impairment should be allowed to retain their functional aids during decontamination. Future research should therefore be carried out to establish the potential advantages and disadvantages of allowing people to retain functional aids during decontamination.

How best to provide training for emergency responders

The majority of the guidance documents do not recognise the importance of training for emergency responders on the psychosocial aspects of managing incidents involving mass decontamination. This is likely due to the fact that planning and preparation for mass decontamination has traditionally focused on the technical aspects involved in the decontamination process, with a tendency to overlook psychosocial issues, such as likely public behaviour and strategies for effective communication with members of the public (13). Where likely public behaviour has been considered within guidance documents, there has been a reliance on assumptions from traditional crowd behaviour theories, which emphasise the likelihood of ‘mass panic’. Evidence from interviews with emergency responders is in line with this, in showing that emergency responders currently receive very little training on likely public behaviour, or how to communicate effectively with members of the public, during incidents involving mass decontamination (69).

The review shows that it is important to design decontamination guidance documents for emergency responders so that psychosocial issues are embedded throughout, and are not an add-on to be considered once the technical aspects of decontamination have been perfected. However, given the apparent reliance on control management strategies and lack of emphasis on communication, or other psychosocial aspects, it is likely that responders will need more than updated guidance in order to fully understand the psychosocial factors associated with decontamination. The available evidence suggests that psychosocial training for emergency responders will be necessary in order to embed an understanding of the psychosocial aspects of managing mass decontamination. Such training should include how to communicate effectively with members of the public during decontamination (13 ^,^ 22 ^,^ 23 ^,^ 24 ^,^ 25 ^,^ 26 ^,^ 57 ^,^ 69), training on the importance of respecting public needs (e.g. privacy, modesty) during decontamination (22 ^,^ 24 ^,^ 25), and training on how to identify and manage those who are at increased risk during decontamination (30). One interesting study by Monteith (2013) (57) revealed that when emergency responders saw a casualty volunteer in distress, their attention quickly moved from a focus on the technical aspects of decontamination, towards a focus on displaying compassion and empathy towards those affected. This suggests that training for emergency responders on the psychosocial aspects of mass decontamination should include information on how members of the public are likely to experience the process, and emphasise the fact that without effective management, decontamination is likely to be embarrassing and distressing for those involved. There are therefore several suggestions for content which should be included in psychosocial training for emergency responders. However, there is very little evidence to suggest what form such training should take and, as such, this is an area where further research is required.

Pre-incident public education

Evidence suggests that pre-incident public education about what actions to take during incidents involving the release of a hazardous chemical could reduce the time needed for people to take initial actions (e.g. evacuation, disrobe) (29 ^,^ 54 ^,^ 58), which could save lives. Available evidence is limited, but that which is available suggests that the provision of pre-incident public education materials may be problematic (people may not want to engage with the material prior to an incident, or may not remember the information when they need it) (88). Hildebrand & Bleetman (2007) (88) carried out a study to examine the effectiveness of two public education booklets distributed to citizens in the UK and Israel. They found that only 33% of participants in the UK and 22% of the participants in Israel remembered reading the booklet. This therefore led to low intention to follow advice, likely because most participants were unaware of Government guidance on recommended actions (having not read the leaflet). This therefore suggests that a public education campaign which simply involves sending an information leaflet to households may not be effective for raising public awareness about actions to take during incidents involving CBRN agents. However, other evidence suggests that engaging members of the public in a more active way (for example, as participants in emergency preparedness field exercises) may help them to understand what actions they should take during a CBRN incident, and an incident involving mass decontamination specifically (29). Taylor et al. found that participants who took part in an exercise involving mass decontamination reported that they felt they would be more prepared if a real incident of this type were to occur, and that it would help them to know what actions to take. Raising public preparedness for an incident involving decontamination is likely to be crucial in order to save lives, as there will inevitably be a delay before specialist teams and resources arrive at the scene (58).

The positive participant response following decontamination exercises suggests that it is possible to engage members of the public in thinking about and preparing for incidents involving decontamination, prior to an incident occurring. However, it is not practical to suggest that all, or even most, of the population would be able to take part in such drills, and evidence suggests that simply sending out information materials is unlikely to have much impact on public preparedness (88). Future research should therefore examine other potential ways to educate members of the public. Suggested options for raising public awareness include passing on information to members of the public through local messengers, providing examples of previous large-scale CBRN incidents, and utilising organised events (e.g. sports events, adult and teen education programmes) to share this information (54). Raising awareness and preparedness for incidents involving mass decontamination among members of the public will ensure that people are able to take protective actions immediately, and will not need to wait for specialist responders and equipment. This could result in lives being saved, and as such should be a priority for future research. Given the potential benefits of successfully providing pre-incident public education, and the fact that evidence relating to this is currently very limited, this is an area which definitely requires further research.

**Limitations**

A limitation of the research presented here is that the review is limited to published guidance documents, and is therefore not exhaustive. While it is likely that public-facing documents will contain a greater emphasis on communication, and less emphasis on public control strategies, this is not necessarily the case. The authors would welcome a signpost to sources of guidance or evidence of ‘state of the art’ practice that is not reflected in this review.

## Conclusion

The review of available research evidence reveals that effective communication and respect for casualties’ needs will be essential in ensuring that decontamination runs smoothly and efficiently. However, the review of guidance documents reveals that the findings from available research have not been used to inform decontamination guidance for emergency responders. The success of decontamination interventions will depend on the successful management of casualties through the decontamination process, and as such it is essential that the psychosocial aspects of mass decontamination are not neglected in the pursuit of technical solutions.

## Corresponding Author

Holly Carter: holly.carter@phe.gov.uk

## Data Availability Statement

The information on conducting the literature review is provided within the paper.

## Competing Interest Statement

The authors have declared that no competing interests exist.

## Appendices

**Appendix 1**

**Figure:**
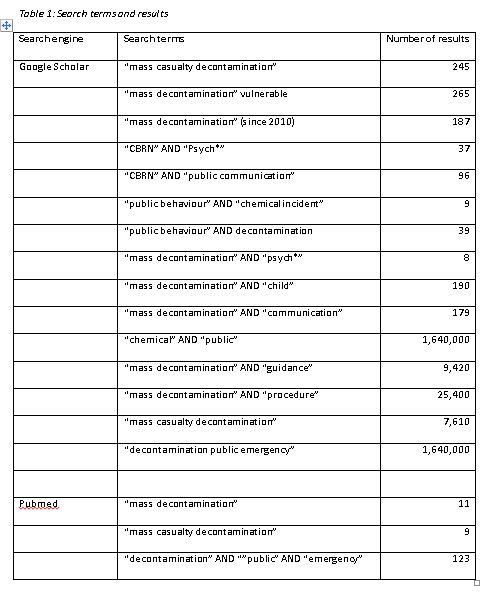
**Table 1: Search terms and results**

**Appendix 2**

**Table 2:** **Summary of key findings relating to psychosocial aspects of incident management for mass casualty decontamination.**

**Appendix 3**

**PRISMA Checklist**
